# The Advent of the COVID-19 Epidemic Did Not Affect Americans’ Endorsement of Moral Foundations

**DOI:** 10.3389/fpsyg.2021.647858

**Published:** 2021-07-28

**Authors:** Irina Vartanova, Kimmo Eriksson, Zeynep Melis Kirgil, Pontus Strimling

**Affiliations:** ^1^Institute for Futures Studies, Stockholm, Sweden; ^2^School of Education, Culture and Communication, Mälardalen University, Västerås, Sweden; ^3^Department of Sociology, Faculty of Social Sciences, Stockholm University, Stockholm, Sweden

**Keywords:** threat, COVID-19, moral foundations, ideology, longitudinal design, conservatism

## Abstract

Prior work has suggested that existential threats in the form of terror attacks may shift liberals’ reliance on moral foundations to more resemble those of conservatives. We therefore hypothesized that endorsement of these moral foundations would have increased when the COVID-19 epidemic became a salient threat. To examine this hypothesis we conducted a longitudinal study with 237 American participants across the liberal-conservative spectrum, in which their endorsement of various moral foundations were measured before and after the advent of the pandemic. We did not find evidence of any systematic change in the endorsement of any moral foundation, neither in general nor specifically among liberals or specifically among those who perceived the greatest threat from COVID-19. We conclude that the threat from the pandemic does not seem to have had any substantial effect on the moral foundations that people rely on. We discuss how this finding relates to other longitudinal studies of the effect of the COVID-19 pandemic on measures related to conservatism.

## Introduction

According to moral foundations theory (Haidt and Joseph, 2007; Haidt, 2012), human morality boils down to a small set of distinct moral foundations: Harm, Fairness, Authority, Ingroup, and Purity (or Sanctity).^1^ These foundations provide reasons for moral judgments. For instance, an act can be judged as immoral because it is harmful, or unfair, or disrespecting of authority, or disloyal, or indecent. According to Haidt and Joseph (2007), these different bases of morality are cultural universals. However, there are important individual differences in the extent to which people rely on specific moral foundations, which are measured using the Moral Foundations Questionnaire (Graham et al., 2009; Graham et al., 2011). This questionnaire asks respondents how relevant various moral arguments corresponding to the five moral foundations are to their moral judgments. A key finding is that individuals’ reliance on moral foundations is linked to their political ideology. While conservatives tend to rely on all five foundations, liberals tend to rely mainly on just two: Harm and Fairness. This difference in what conservatives and liberals regard as relevant foundations of morality is thought to explain major political differences in moral judgments (Haidt, 2012; Koleva et al., 2012; Strimling et al., 2019). To describe this phenomenon, moral foundation researchers group the moral foundations in two categories: Harm and Fairness are referred to as *individualizing* foundations because they are conceived as supporting individual-focused contractual approaches to society, while Authority, Ingroup, and Purity are referred to as *binding* foundations because they are conceived as binding people together into larger groups and institutions (Graham et al., 2011). We can view an individual’s endorsement of individualizing and binding foundations as a key to understanding the moral judgments that person will make. The more people rely on binding foundations, the more conservative we expect their morality to be.

Against this background, it is of great interest to understand the factors that shape an individual’s endorsement of various moral foundations. One such factor suggested by previous research is the role of threat, which has long been thought to be linked to political conservatism (Jost et al., 2003). Two different threats that have been considered are terror attacks and infectious diseases. Several studies have examined whether people’s political preferences and moral attitudes become more conservative after experiencing a terror attack (Bonanno and Jost, 2006; Van de Vyver et al., 2016; Brouard et al., 2018; Castanho Silva, 2018; Choma et al., 2018; Lindén et al., 2018). A couple of studies specifically address the relation between terror and endorsement of moral foundations. Wright and Baril (2013) found in a laboratory experiment that a writing exercise about the September 11 terror attacks increased liberals’ reliance on binding foundations. Of particular interest is a study of Van de Vyver et al. (2016) who surveyed representative samples of the United Kingdom population before and after the 2005 London bombings. They found that, specifically among liberals, endorsement of in-group loyalty (to Britain) had slightly increased and that endorsement of fairness (equality for all groups in Britain) had decreased. These papers do not report effect sizes, but it is clear from figures that the shifts among liberals are only a fraction of the full distance between liberals and conservatives. In sum, prior research supports that existential threats in the form of terror attacks may shift the moral foundation of liberals to (somewhat) more closely resemble the moral foundations of conservatives.

It has been suggested that disease threat too could affect moral foundations, and this notion is supported by some correlational evidence that people in societies exposed to greater disease threat rely more strongly on binding foundations whereas the reliance on individualizing foundations is not related to disease threat (Van Leeuwen et al., 2012). We are not aware of any prior studies on this link that use experiments or natural experiments. When the COVID-19 pandemic hit the world, we realized that we could use some existing data we had recently collected on moral foundations as a first wave in a longitudinal study of the effect of the COVID-19 threat on people’s endorsement of moral foundations. We had the following expectations based on prior work. First, directly derived from the work of Van Leeuwen et al. (2012), we expected that after the emergence of the pandemic, people would more strongly endorse binding foundations but not change their endorsement of individualizing foundations. Second, we expected to replicate the finding in prior work (Wright and Baril, 2013; Van de Vyver et al., 2016) that threat has a greater effect on moral foundations among liberals than among conservatives, making liberals more similar to conservatives. Third, we expected that individual variation in the change in moral foundations would be related to variation in threat perceptions, so that those who perceive greater threat from COVID-19 will have changed their moral foundations more. Such evidence seems crucial to enable conclusions that it is specifically the perceived disease threat that is the cause of shifts in reliance on moral foundation.

Note that although no prior experimental or longitudinal work has examined the effect of disease threat on moral foundations, there are several such studies of how various related constructs (e.g., social conservatism, political conservatism, right-wing authoritarianism, social-dominance orientation, gender stereotypes, and sexual prejudice) were affected by the COVID-19 pandemic (Fischer et al., 2020; Golec de Zavala et al., 2020; Karwowski et al., 2020; Rosenfeld and Tomiyama, 2021) and the 2014 Ebola outbreak in the United States (Beall et al., 2016; Inbar et al., 2016; Schaller et al., 2017; Tiokhin and Hruschka, 2017). These studies have yielded a mix of small effects and null effects. We make a more detailed examination of these previous results in the Discussion to fit our own study into the bigger picture.

## Methods

The Moral Foundations Questionnaire, or MFQ, measures reliance on five moral foundations: harm, fairness, authority, ingroup loyalty, and purity (Graham et al., 2009, 2011). The original MFQ has two parts. One part consists of “relevance items,” three for each moral foundation, asking participants to rate how relevant various moral concerns are to their moral judgments. The other part consists of “judgment items,” three for each moral foundation, asking participants to rate how much they agree with various moral judgments.

During 2019 and early 2020, our lab had run a series of small and unpublished studies on MTurk in which we tried out variations of the MFQ for reasons unrelated to the current project. When COVID-19 developed into a serious pandemic, we decided to use these data as the first wave of a longitudinal study. Participants were anonymous to us, but the system offers the possibility of reinviting participants. This presented a unique opportunity for conducting a longitudinal study of moral foundations covering both the time before and the time after the arrival of the epidemic. Between mid-March and early May 2020 we therefore invited the same participants to take the moral foundation survey again, with a few additional questions relating to the perceived threat from COVID-19.

### Versions of the MFQ Used in the Study

For reasons unrelated to the current project, our lab had run nine different versions of the MFQ. Table 1 provides an overview of the nine versions of the MFQ that we used. Item numbers refer to the list of relevance items in the original MFQ, which read as follows: When you decide whether something is right or wrong, to what extent are the following considerations relevant to your thinking? Whether or not (1) someone suffered emotionally, (2) some people were treated differently than others, (3) someone’s action showed love for his or her country, (4) someone showed a lack of respect for authority, (5) someone violated standards of purity and decency, (6) someone was good at math, (7) someone cared for someone weak or vulnerable, (8) someone acted unfairly, (9) someone did something to betray his or her group, (10) someone conformed to the traditions of society, (11), someone did something disgusting, (12) someone was cruel, (13) someone was denied his or her rights, (14) someone showed a lack of loyalty, (15) an action caused chaos or disorder, and (16) someone acted in a way that God would approve of.

**TABLE 1:** Different versions of the MFQ questionnaire included in the study.

**Ver.**	**Included items**	**Time 1**	**Time 2**	**N_*time 1*_**	**N_*time 2*_**	**N_*covid*_**
1	Full MFQ with both relevance and judgments items.	18–19 Sep 2019	12–15 Mar 2020	99	73	73
2	Relevance items 1–5, 7–11 (two items for each MF)	13 Feb 2020	29 Apr 2020	35	22	14
3	Relevance items 3–5, 9–11 (two items for each binding MF)	13 Feb 2020	29–30 Apr 2020	28	20	16
4	One random item for each MFs: 1 or 7, 2 or 8, 3 or 9, 4 or 10, 5 or 11	14 Feb 2020	29–30 Apr 2020	28	23	15
5	Four relevance items from binding MFs: 4, 5, 10, 14	14 Feb 2020	28–30 Apr 2020	32	19	11
6	Relevance items 1, 2, 4, 5, 7–11, 14 (two items for each MF)	19–20 Feb 2020	29 Apr to 7 May 2020	28	24	20
7	Relevance items 4, 5, 9–11, 14 (two items for each binding MFs)	19–20 Feb 2020	29 Apr to 4 May 2020	30	19	14
8	Relevance items 1, 2, 4, 5, 14 (one item for each MF)	19–20 Feb 2020	2–10 May 2020	28	19	19
9	One item for each binding MF: 4, 5, 14	19–20 Feb 2020	29 Apr to 3 May 2020	24	18	15

*N_covid_ indicates the number of participants who completed the COVID-19 specific block of items at time 2.*

### Sample

In the first wave, a total of 332^2^ Mturk workers completed one of the MFQ questionnaire versions. See Table 1 for the exact number of participants taking each version. In the second wave, in which data were collected between March 12 and May 10, 2020, participants were invited to retake the same version of the survey,^3^ with the addition of some COVID-19 specific items. The final sample comprises 237 Mturk workers who participated in both waves (45% women; average age 42 years with a standard deviation of 11 years; 52% self-identifying as liberals and 32% as conservatives).

### Measures

#### Change in Moral Foundations

MFQ items have a response scale from 0 to 5. For each participant we calculated the score for each moral foundation by averaging the available items that correspond to that foundation. The change in the endorsement of a given moral foundation was then calculated as the difference between the second wave and first wave ratings. In the subsample, where the full MFQ was used, the change score for a given moral foundation is thus based on change in six items. The other versions used fewer items and these change scores may therefore have lower reliability. For this reason we report results both for the full sample (but “part MFQ”) and for the “full MFQ” subsample.

#### Perceived Threat From COVID-19

The perceived threat from COVID-19 was measured by three items on how much participants worry about the coronavirus (5-point scale from 1 = Not worried at all to 5 = Extremely worried), how often they think about how the coronavirus might affect them (from 1 = Never to 5 = All the time), and how much they think other people worry about the coronavirus (from 1 = Way too much to 5 = Way too little). An aggregated index of the COVID-19 related perceived threat was computed and standardized (α = 0.82).

The survey also included items on whether the participant knew someone personally who was quarantined (21% did) or whether the participant knew someone personally who was a confirmed case (18% did). As we expected, these participants perceived a somewhat higher threat on average.

#### Ideology

Participants were asked where they would place their political views on a 7-point scale between extremely liberal and extremely conservative, with libertarian as an additional option. We coded those who selected 1–3 on the scale as liberals, 4 as moderates, 5–7 as conservatives, and excluded three libertarians. 14 participants reported another political affiliation (conservative, moderate, or liberal) in the second than in the first wave. We use the self-identification from the first wave when we conduct analysis by separate ideological groups.

## Results

To start with we validate our data by showing that they replicate the basic findings of Graham et al. (2011). These basic findings are that, compared to conservatives, liberals rely somewhat more on harm and fairness and much less on authority, ingroup loyalty, and purity. For this analysis we use the data from the first wave. Table 2 shows mean scores among liberals, moderates, and conservatives in our sample together with the corresponding mean scores in the original study of Graham et al. (2011). Prior findings generally replicated well. We conclude that our sample is not atypical.

**TABLE 2:** Mean relevance scores for different moral foundations among liberals, moderates, and conservatives, with corresponding mean values from Graham et al. (2011) within parentheses.

**MF**	**Liberal**	**Moderate**	**Conservative**
Harm (*SD* = 1.13)	3.98 (3.93)	3.83 (3.68)	3.46 (3.48)
Fairness (*SD* = 1.00)	4.05 (4.04)	3.95 (3.77)	3.79 (3.44)
Authority (*SD* = 1.26)	2.09 (1.88)	2.46 (2.37)	2.82 (2.81)
Ingroup (*SD* = 1.20)	1.84 (2.06)	2.30 (2.56)	2.44 (3.03)
Purity (*SD* = 1.50)	1.62 (1.44)	2.22 (2.09)	2.69 (2.88)

Attrition should also be considered. 29% of first-wave participants did not take part in the second wave of the study. The first-wave responses to moral foundations among these dropouts were not significantly different from other participants for any item, all *p* > 0.11. We conclude that attrition was not a major concern for our study.

### Change in Endorsement of Moral Foundations

**FIGURE 1:**
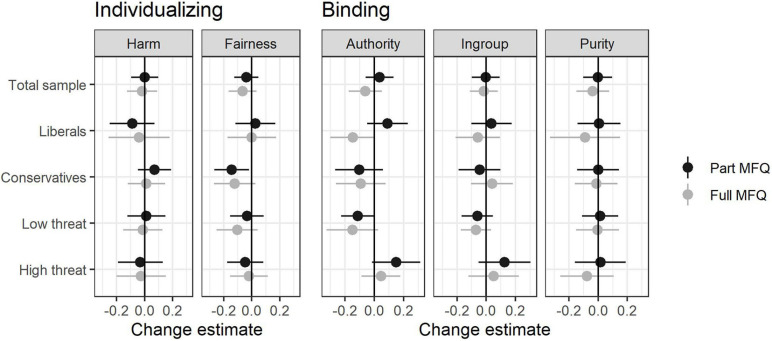
Mean change scores for endorsement of individualizing (left) and binding (right) moral foundations in the full sample, including those who completed a part MFQ (black), and in the subsample that completed the full MFQ (gray), and in each of four subsamples defined by ideology and perceived threat from COVID-19. Error bars indicate 95% confidence intervals.

We report change in two different ways. Figure 1 shows, for each moral foundation, the mean change with a 95% confidence interval. Table 3 instead reports the effect size measure known as Cohen’s *d*_*av*_, that is, mean change standardized by the average standard deviation across the two waves (Cumming, 2013). Table 3 also reports sample sizes. As a robustness check, Figure 1 and Table 3 report results both in the full sample and in the subsample that completed the full MFQ.

**TABLE 3:** Mean increase or decrease (negative values) in standardized moral foundations scores (*d*_*av*_) in the full sample (top half) and in the subsample that completed the full MFQ (bottom half).

	**Total sample**		**Conserv.**		**Liberals**		**Low threat**		**High threat**	
**MF**	***d***	***n***	***d***	***n***	***d***	***n***	***d***	***N***	***d***	***n***
Harm	0.00	161	0.06	57	−0.10	79	0.01	68	−0.03	73
Fairness	−0.04	161	−0.14	57	0.03	79	−0.04	68	−0.05	73
Authority	0.03	237	−0.08	77	0.08	123	−0.09	101	0.12	96
Ingroup	−0.00	237	−0.04	77	0.03	123	−0.05	101	0.11	96
Purity	−0.00	237	−0.00	77	0.00	123	0.01	101	0.01	96
Harm	−0.02	73	0.01	38	−0.05	26	−0.01	40	−0.03	33
Fairness	−0.09	73	−0.16	38	0.00	26	−0.15	40	−0.02	33
Authority	−0.06	73	−0.11	38	−0.14	26	−0.15	40	0.04	33
Ingroup	−0.02	73	0.05	38	−0.05	26	−0.07	40	0.05	33
Purity	−0.03	73	−0.01	38	−0.08	26	−0.00	40	−0.06	33

Our first research question was whether the COVID-19 pandemic would lead to an increase in the endorsement of binding foundations but not in the endorsement of individualizing foundations. To answer this question we consider the mean changes in the entire sample, which are given in the first columns of Table 3 (“Total sample”) and the first row of Figure 1. These results indicate a negative answer to the question. Specifically, there was no evidence of an increase in endorsement of any moral foundation.

Our second question was whether there would be a specific shift among liberals toward more conservative moral foundation scores. To answer this question we consider the mean changes among liberals and conservatives separately, which are given in the second and third columns in Table 3 and the second and third rows of Figure 1. Note that change among liberals was negligible (*d*_*av*_ ≤ 0.1) for all moral foundations except for authority, which instead yielded conflicting results depending on sample (part MFQ or full MFQ). Thus, there was no consistent evidence for a shift in moral foundation scores specifically among liberals.

Our third expectation was that changes would be clearer among those who perceived greater threat from COVID-19. We therefore performed a median split on the measure of perceived threat at the value of 2.7, which is close to the midpoint of the three items measured on the 5-points scale. The sample was thus divided into two subsamples representing “low perceived threat” and “high perceived threat”. See the last two columns in Table 3 and the last two rows in Figure 1. Note that even in the high threat sample most changes were negligible (*d*_*av*_ ≤ 0.1) and not significant, with slightly larger changes only for the authority and ingroup foundations, and then only in the full sample and not in the full MFQ subsample.^4^

## Discussion

This study was conceived in March 2020, when the COVID-19 pandemic threat had just arrived. New data was collected (between mid-March and early May 2020) and compared with data on the same participants collected earlier (in mid-September 2019 or mid-February 2020). The study was motivated by expectations on how this threat could affect people’s endorsement of specific moral foundations. Based on prior research linking the historical prevalence of infectious diseases to endorsement of binding moral foundations (Van Leeuwen et al., 2012), we expected that binding moral foundations would be activated by the emergence of a novel infectious disease threat. Moreover, we expected changes to be driven by liberals in order to replicate findings from prior research on the effect of terror threat (Wright and Baril, 2013; Van de Vyver et al., 2016). Finally, based on the notion that changes would be triggered specifically by the perceived threat, we expected changes to be larger among those who perceived greater threat from COVID-19. The results of our study met none of these expectations. The data indicate that the threat associated with the pandemic had no clear effect on endorsement of binding (or individualizing) moral foundations, not even among liberals or among those who perceived the greatest threat from COVID-19.

We recognize that our sample size is not very large. One should therefore consider whether there could still be a sizable effect of COVID-19 on moral foundations in the population, which was masked by sampling error. The confidence intervals give the answer to this question. They show that it would have been unlikely to obtain our results if there were a substantial effect in the population as a whole. For example, consider the moral foundation of purity. According to the original formulation of moral foundations theory, the purity foundation evolved specifically as a pathogen-defense system (Haidt and Joseph, 2007). From that theoretical perspective, the purity foundation would be especially likely to be activated by an emerging threat of infectious disease. Yet, the high end of the confidence intervals we obtained indicate that endorsement of purity increased at most on the order of 0.1 scale points in the sampled population. Thus, our data indicate that, to the extent that COVID-19 had any effect on the moral foundations of the population in the United States, this effect was not of any substantial size.

We shall now compare our findings to what related studies have found. Some of these studies rely on a theoretical division of political ideology measures into two broad dimensions (Claessens et al., 2020). This theory distinguishes between those ideology measures that are related to cooperation (e.g., economic conservatism, social dominance orientation, individualizing moral foundations) and those related to conformity (e.g., social conservatism, right wing authoritarianism, binding moral foundations). The theory predicts that only the conformity dimension is affected by threat whereas the cooperative dimension should be affected by increased competition (Duckitt and Sibley, 2009). In the below discussion of prior findings, we have clarified for each result whether it belongs to the cooperation dimension or the conformity dimension. Note that general measures of political leaning from conservative to liberal are ambiguous as it could be interpreted either as social or economic conservatism.

### Comparison to Other Studies of the COVID-19 Pandemic

The COVID-19 pandemic has inspired several studies related to ours, generally producing small effects on conformity related measures and no effect on measures related to cooperation. One study used a randomized experiment in Poland and the United States to examine whether making the threat of COVID-19 salient affected social conservatism (Karwowski et al., 2020). Although the threat elevated participants’ anxiety, no effect of threat salience on social conservatism (conformity) was found in either country.

Three studies used a longitudinal design with pre- and post-pandemic measures, similar to ours. A study in Poland (Golec de Zavala et al., 2020) found small increases (*d*_*av*_ around 0.15) in right-wing authoritarianism (conformity) and sexual prejudice (conformity), and no changes in social-dominance orientation (cooperation) or political conservatism (ambiguous). A study in the United Kingdom (Fischer et al., 2020) found a very small increase (*d*_*av*_ around 0.05) in right-wing authoritarianism (conformity) and no change in social-dominance orientation (cooperation). The increase in right-wing authoritarianism was only very weakly related to the perceived threat from the pandemic (explaining only 5% of the variance) and not related to actual exposure. A study in the United States (Rosenfeld and Tomiyama, 2021) found a very small increase (*d*_*av*_ around 0.1) in endorsement of gender stereotypes (conformity) and no change in political conservatism (ambiguous). The increase in gender stereotypes was not related to perceived threat from the pandemic.

In sum, studies of the effect of the COVID-19 epidemic on measures related to conservatism show a consistent overall pattern: there is often a small shift in values related to conformity and no shift in values related to cooperation. To the extent that a shift is observed, it is only weakly, if at all, related to people’s perceived threat, thus making it doubtful whether any observed shifts should at all be attributed to a psychological effect of the threat. The present study diverges slightly from this pattern in that we did not detect an effect either on cooperative values or on conformity values. However, the small effect suggested by the previous studies is within the confidence interval of our results.

### Comparison to Studies of the Ebola Outbreak

Some related studies were conducted in connection with the 2014 Ebola outbreak in the United States to examine if it led to increased conservatism. One group of researchers reported an increase in support for Republican candidates (ambiguous) after the onset of the epidemic, especially in already conservative states (Beall et al., 2016; Schaller et al., 2017), but other researchers found the evidence to be inconclusive, as the observed shift could also be attributed to temporal autocorrelation (Tiokhin and Hruschka, 2017). A study of implicit attitudes toward homosexuals (conformity) observed a small discontinuity in the trend just after the Ebola outbreak but a *t-*test did not reveal any actual change (Inbar et al., 2016). Thus, in line with the present study, findings from the Ebola outbreak yielded little conclusive evidence that the emergence of a disease threat caused an increase in conservatism.

### Comparison to Other Studies of Real World Threats

There has been a number of studies on the effect of other threats, especially terrorism, on conservative values. In a meta-analysis, Jost et al. (2017) looked at 59 studies of how threatening real world events had affected conservatism in a broad sense (including preferences for conservative leaders, parties, opinions, values, orientations, and policies). Positive effects were found in roughly half of the studies (35 out of 59) and on average the effect size was negligible to small (*r* = 0.07–0.14). The variance in effect size was higher than one would expect if the underlying effects sizes would have been the same, suggesting that the effect of real-world threat on conservative values is moderated by factors such as the specific kind of outcome measure (the study did not distinguish between measures related to cooperation and measures related to conformity) and the specific kind of threatening event.

From this review we conclude that our null findings fit well within the range of effects on measures of conservatism found in prior studies of real world threats. Some studies find positive effects and some do not. It may be that, compared to some other real world threats like terrorists, epidemics are perceived to have less agency and therefore differ in their psychological impact. It may also be that reliance on binding moral foundations are more inert than more explicit measures of conformity-related values. It is not clear by what process respondents to the MFQ arrive at estimates of how relevant a moral foundation is to their moral judgments, but it may well include respondents performing some kind of review of prior judgments they have made, in which shifts in responses to MFQ may display some time lag.

### Limitations

Several limitations should be acknowledged. Because the COVID-19 pandemic was not foreseen, the study was not pre-planned. Our sample was determined by the set of relevant data collections that we happened to have run as pilot studies for another project. The sample was therefore not very large, nor nationally representative, but these limitations are attenuated by the longitudinal design and the fact that both liberals and conservatives were well represented in the sample. The study was conducted with participants from the United States, which is a country where a conservative president downplayed the threat of COVID-19 (Rutledge, 2020). However, liberals still tended to believe in the threat, yet we found no clear effect on the moral foundations of liberals nor did we find an effect among those feeling threatened by COVID-19.

Some of our data for the first wave of the study were collected in mid-February, 2020. This was well before COVID-19 was declared a pandemic but after it had started spreading. Thus, it is possible that some people had already started to worry at that point. However, our findings remained the same when we only considered the “full MFQ” participants who took the first part months before the outbreak of COVID-19. Participants took the second wave of the study between March 12 and May 10, a period during which the sense of threat increased (Clinton et al., 2021). However, concerns about COVID-19 were high already on March 12 according to Google Trends.^5^ Note that our data speak only to the effect of the arrival of the pandemic and not to its effects in the longer term.

## Conclusion

In this study, we did not find an effect of the existential threat posed by the COVID-19 pandemic on people’s endorsement of moral foundations. In contrast to the predictions, binding foundations did not change more than individualizing foundations and people who felt very threatened did not change their moral foundation more than people who felt little threat. Related studies of the effect of COVID-19 on various measures of conservative values have yielded a mix of null effects and small effects (typically for measures related to conformity). Importantly, no study has found a substantial relation between changes in conservative values and concerns about the epidemic. Thus, the bigger picture seems to be that there have been no, or at least negligible, effects on individuals’ conservative values from the threat posed by COVID-19. Our study fits well into this bigger picture. On a societal level, some studies indicate there may have been a small shift in some conformity-related values; this shift could be related to some society level process caused by the pandemic or it could just be coincidental with the pandemic. This may become clearer as more data on shifts in political values become available from polling institutes. It should be noted that this research has only measured the short-term effects of the pandemic. As the pandemic is now in its second year and still ongoing, long-term consequences may be addressed in future research.

## Data Availability Statement

The datasets presented in this study can be found in online repositories. The names of the repository/repositories and accession number(s) can be found below: https://github.com/irinavrt/mfq-under-threat.

## Ethics Statement

Ethical review and approval was not required for the study on human participants in accordance with the local legislation and institutional requirements. The patients/participants provided their written informed consent to participate in this study.

## Author Contributions

IV and PS designed the study. IV collected the data. IV and ZK analyzed the data with input from PS and KE. ZK and IV wrote the methods section. KE and IV wrote the rest of the manuscript. KE, PS, and IV revised the manuscript. All authors contributed to the article and approved the submitted version.

## Conflict of Interest

The authors declare that the research was conducted in the absence of any commercial or financial relationships that could be construed as a potential conflict of interest.

## Publisher’s Note

All claims expressed in this article are solely those of the authors and do not necessarily represent those of their affiliated organizations, or those of the publisher, the editors and the reviewers. Any product that may be evaluated in this article, or claim that may be made by its manufacturer, is not guaranteed or endorsed by the publisher.

## Footnotes

1 Sometime labels that emphasize the bipolarity are used instead: Care/Harm, Fairness/Cheating, Loyalty/Betrayal, Authority/Subversion, Sanctity/Degradation.

2 Some Mturk workers participated in more than one version. In those cases we used only the earliest version for each participant.

3 Participants who originally took version 4, where items were randomized, received version 2 in the second wave; this version includes the same items but without randomization.

4 We also checked that the results hold for direct experience with the pandemic, even though the small sample size (*n* = 72) makes the estimates imprecise and, therefore, inconclusive. Using the subsample of participants who knew someone with a confirmed COVID-19 case, there was no significant shift in their moral foundations (*d*_*av*_ was 0.10 for Harm, 0.00 for Fairness, 0.01 for Purity, 0.08 for Ingroup, and 0.14 for Authority, all *p* > 0.13). The somewhat larger increase for Authority occurred mainly among the participants with low perceived threat (*d*_*av*_ = 0.2, *n* = 35) while among those with high perceived threat the change was very small (*d*_*av*_ = 0.06, *n* = 26), making it unlikely that it is an effect of threat.

5
https://trends.google.com/trends/explore?date=2020-02-01%202020-05-31&geo=US&q=%2Fg%2F11j2cc_qll
